# The forensic pathological analysis of sport-related sudden cardiac death in Southern China

**DOI:** 10.1080/20961790.2017.1319785

**Published:** 2017-05-31

**Authors:** Yeda Wu, Mei Ai, Adham Sameer A. Bardeesi, Liyong Zhang, Qiuping Wu, Kun Yin, Jingjing Zheng, Da Zheng, Lei Huang, Lunwu Xu, Jianding Cheng

**Affiliations:** a Department of Forensic Pathology, Zhongshan School of Medicine, Sun Yat-Sen University, Guangzhou, China;; b Forensic Science Center of Waston, Guangzhou, China;; c Branch Office of Yanping, Public Security Bureau of Nanping, Nanping, China

**Keywords:** Forensic sciences, forensic pathology, sport, sudden death, cardiac, coronary disease, cardiomyopathies

## Abstract

Studies regarding sport-related sudden cardiac death (SCD) mainly focus on competitive athletes; similar data are rare in the general population, especially in China. We conducted a retrospective study (from September 1998 to August 2013) to investigate the aetiological distribution and epidemiological features of sport-related SCD in Southern China. Selections of cases are based on details, and two subgroups were established: one was the sport-related SCD group, and the other was the disease-free accident victims group which was matched with the sport-related SCD group in gender, age and year of death. Among the 3770 sudden-death cases, 1656 cases were SCD cases. A total of 65 cases (57 males) out of 1 656 SCD cases were sport-related. The age range of the 65 sport-related SCD cases was from 12 to 68 years old with a mean (35.92 ± 14.23) years old. Only two of these cases were competitive athletes. The most common circumstances of the 65 sport-related SCD cases were heavy physical labour (46.15%) and running (30.77%). The three leading forensic diagnoses were the coronary atherosclerotic disease (CAD, 28 cases), cardiomyopathy (CM, 14 cases) and sudden unexplained death (7 cases). CM was the most common forensic diagnosis in those ≤35 years old, while CAD was the most common one in those >35 years old. Left anterior descending in which atherosclerotic plaques was most commonly found was the principal artery branch associated with sport-related SCD. There was a statistically significant difference in the weight of hearts between the 65 sport-related SCD cases and 65 diseases-free accidental cases. This study highlights the need to attract public attention to sport-related SCD and to issue a prevention strategy to the public, and to make the SCD-related genetic sequencing a routine tool in both forensic pathological examination and clinic screening.

## Introduction

Sudden cardiac death (SCD) is a sudden, unexpected and rapid natural death due to a cardiac disease, occurring within 1 h from the onset of symptoms such as chest pain, in individuals without any prior condition that appears fatal [1]. Due to its transience and unpredictability, people who manifest the fatal symptoms of SCD cannot be rescued in time, causing a deep sorrow for their relatives, even for the society. The annual incidence of SCD in China was 41.8/100 000 reported by Hua et al. in 2009 [2]. Due to China’s large population, the number of SCD cases is extremely high. To date, SCD has become a severe public health problem, and developing comprehensive preventive strategies against SCD is a pressing matter.

There are so many triggers, which can cause SCD, such as physical activity, mental stimulation, overeating, etc. As for physical activity, it is well accepted that regular physical activities play a positive role in health, especially in the cardiovascular system. On the contrary, physical exertion, including intensive exercise and heavy physical activity, is associated with cardiac fatigue [3] and increases the risk of SCD [4]. Nowadays, public and research attention mainly focuses on sport-related SCD in young competitive athletes, and several studies regarding this have been performed in different countries [5–7]. Table 1 displays the summary of current studies regarding sport- related SCD. However, there are few papers on sport- related SCD for physical activities performed during daily leisure-time physical activities. Besides, very few studies are available regarding the cases of heavy physical labour-related deaths.

**Table 1.** Sport-related SD in series.

Authors	Period of study	Area	Population characteristics (case number)	Total number of sport-related SD (case number)	Age (years old)	Mean age (years old)	Male:female	The three leading types of sport (case number)	The leading forensic diagnosis (case number)		
									All subjects	Age ≤35 years old	Age >35 years old
Holst et al. [5]	2000–2006	Denmark	Competitive athletes (15)	SCD (15)	15–35	26.4 ± 6.4	11:4	Soccer (5) Running (5) Handball (2)	SUD (4) ARVC (4)	SUD (4) ARVC (4)	NA
Maron et al. [6]	1980–2006	USA	Competitive athletes (1 866)	SD events (1 866)^a^	8–39	18 ± 5	1 692:174	Football (565) Basketball (405) Soccer (115)	HCM (251)^b^	NA	NA
de Noronha et al. [7]	1996–2008	UK	Amateur sportsmen (107) and professional-athlete level individuals (11)	SCD (118)	7–59	27.9 ± 12.5	113:5	Soccer (44) Running (24) Rugby (11)	MNH (27) ILVH (27)	MNH (26)	ILVH (8)
Allouche et al. [8]	2005–2009	Tunisia	Amateur athletes (32)	SD (32)	15–79	33.16	27:5	Running (13) Football (10) Dance (4)	CAD (9) HCM (9)	HCM (9)	CAD (9)
Marijon et al. [9]	2005–2010	France	Competitive athletes (50) and general individuals (770)	SD (820)	11–75	46 ± 15	777:43	Cycling (251) Jogging (175) Soccer (107)	SUD (619)	NA	NA
Suárez-Mier et al. [10]	1995–2010	Spain	Professional athletes (3) and general individuals (165)	SD (168)	9–69	36.6 ± 15.6	163:5	Cycling (49) Soccer (43) Running (15)	CAD (85)	SUD (19)	CAD (74)
Chappex et al. [11]	1995–2010	Switzerland	Athletes (2) and non-athletes (20)	SCD (22)	11–50	30.5 ± 13.5	18:4	Hiking (5) Swimming (4) Skiing (4)	CAD (6)	MNH (4)	CAD (5)
Present study	1998–2013	China	Athletes (2) and non-athletes (63)	SCD (65)	12–68	35.92 ± 14.23	57:8	Heavy physical labour (33) Running (20) Cycling (5)	CAD (28)	CM (10)	CAD (21)

SD, sudden death; SCD, sudden cardiac death; SUD, sudden unexplained death; ARVC, arrhythmogenic right ventricular cardiomyopathy; HCM, hypertrophic cardiomyopathy; MNH, morphologically normal heart; ILVH, idiopathic left ventricular hypertrophy; CAD, coronary atherosclerotic heart disease; CM, cardiomyopathy; NA, not available.

^a^Including 85 cardiac arrest survivors

^b^Just list the confirmed cardiovascular-events diagnosis

In order to determine the overall sport-related SCD burden, to characterize the features of sport- related SCD, and to identify the risk factors for sport-related SCD in general population, we conducted a retrospective study of 65 sport-related SCD cases, which were identified after analysis of case, comprehensive autopsies and pathological examination in the Medicolegal Expertise Center of Sun Yat-Sen University.

## Methods

### Cases collection

Based on the 3 770 sudden-death cases identified after systematic and thorough autopsies, and histopathological examinations in the Medicolegal Expertise Center of Sun Yat-Sen University from September 1998 to August 2013, 1 656 cases were identified as SCD according to the definition that deaths occur within 1 h of the sudden loss of consciousness due to various cardiovascular diseases, whereas deaths due to non-cardiac conditions, such as injuries, poisonings, epilepsy, acute pulmonary embolisms and allergies, were excluded. Definitions and main features for specific cardiac pathologies have been previously described [12]. Sudden unexplained death (SUD) is a diagnosis of exclusion, defined as a structurally normal heart with no evident abnormality on macroscopic and histological evaluation, and a negative result for toxicology screening. After assessing and analysing the case details, a subgroup (65 cases, including 2 athletes died during intensive sport training and 63 individuals died during daily sport activities) was established as sport-related SCD group, of which the death occurred during or within 1 h after sports activities. These cases that victims died during or within 1 h after heavy weightlifting work (such as handling and loading) were defined as heavy physical labour. Seventeen sprint cases and three long-distance cases were described as running. Yoga exercise was also included to describe the circumstance of sport-related SCD cases although it is controversial as to whether it is a kind of sport or not.

The inclusion criteria for the 65 cases of accidental events included death due to electrical shock (12 cases), mechanical asphyxia (38 cases), intoxication (13 cases) and anaphylaxis (2 cases). All studies and data collection were conducted in strict accordance with ethics guidelines of the Zhongshan School of Medicine of Sun Yat-Sen University.

### Statistical methods

Subjects were divided into two groups by their age at death: ≤35 years old and >35 years old [7]. Data on age, gender, circumstance of death, cause of death, height, heart weight and the circumference of all heart valves were obtained from death report. Coronary stenosis was divided into four grades according to lumen area reduction in the segments with the most severe narrowing. Grade I lesions: luminal area reduced by 1%–25%; grade II lesions: luminal area reduced by 26%–50%; grade III lesions: lumen area reduced by 51%–75%; grade IV lesions: lumen area reduced by 76%–100%. A total of 65 sport-related SCD death cases were analysed by SPSS 13.0 software (SPSS, Inc., Chicago, IL, US). Pearson Chi-square tests or Fisher’s exact tests were used to analyse the differences in the proportions of the main pathogenic causes and the differences in the distributions of pathological changes in the three branches of coronary artery according to age. The Kolmogorov–Smirnow test was used to check the normal distribution in each variable. The *T*-test or Mann–Whitney *U*-test was used to analyse the difference between 65 sport-related SCD cases and 65 disease-free accident events cases. Test results with two-tailed values of *P <* 0.05 were considered statistically significant.

## Results

### The demographics of sport-related SCD

**Figure 1.**
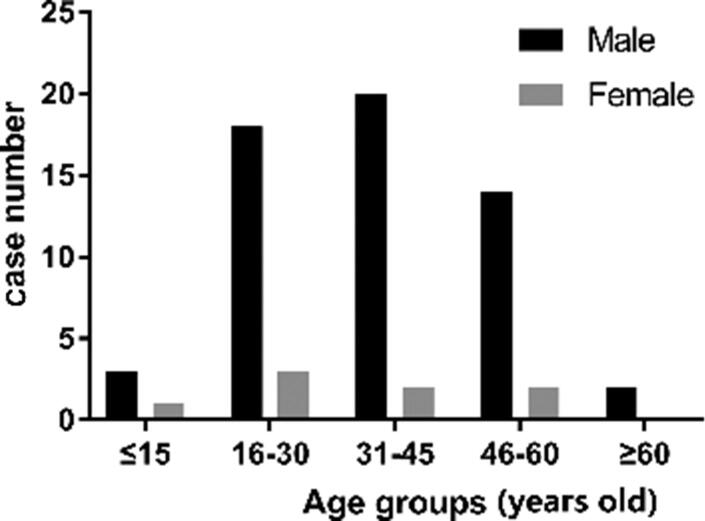
The age and gender distributions of sport-related sudden cardiac death cases.

A total of 65 out of 1 656 SCD cases were identified as sport-related SCD cases. The average age of 65 sport-related SCD cases was (35.92 ± 14.23) years old, and the median age was 37 years old. Among the 65 sport-related cases, 57 cases were male. The male-to-female ratio is 7.1:1. When all the enrolled 65 sport-related cases were divided into five age groups (<15 years old, 16–30 years old, 31–45 years old, 46–60 years old and >60 years old), the age groups of 31–45 years old in male (20 cases) and 16–30 years old in female (3 cases) were predominant in sport-related SCD. The age and gender distributions are illustrated in Figure 1.

### The circumstances of death in sport-related SCD

Most of the sport-related SCD were observed during heavy physical labour (50.77%), running (30.77%) or cycling (7.69%). The circumstances of death in sport- related SCD are illustrated in Table 2.

**Table 2.** The circumstances of death in sport-related sudden cardiac death.

The circumstances of death	Case number	%
Heavy physical labour	33	50.77
Running	20	30.77
Cycling	5	7.69
Basketball	3	4.62
Football	1	1.54
Skating	1	1.54
Yoga	1	1.54
Swimming	1	1.54
**Total**	**65**	**100**

### The forensic diagnoses of sport-related SCD cases

Based on the comprehensive autopsy examinations, the forensic pathological diagnoses of 65 sport-related SCD cases are illustrated in Table 3. Coronary atherosclerotic heart disease (CAD) was the leading diagnosis of sport-related SCD cases followed by cardiomyopathy (CM) and SUD. The cases of CM included five cases of arrhythmogenic right ventricular cardiomyopathy (ARVC), four cases of dilated cardiomyopathy (DCM), three cases of restrictive cardiomyopathy (RCM) and two cases of hypertrophic cardiomyopathy (HCM). The cases of cardiac conduction system diseases primarily involved fatty infiltration or fibrosis of the cardiac conduction system.

**Table 3.** The distributions of forensic pathological diagnoses between different age groups of 65 sport-related sudden cardiac death cases (case number).

Forensic pathological diagnoses	Age ≤35 years old	Age >35 years old	Total
Coronary atherosclerotic heart disease	7	21	28
Cardiomyopathy			
Arrhythmogenic right ventricular cardiomyopathy	4	1	5
Restrictive cardiomyopathy	3	0	3
Dilated cardiomyopathy	2	2	4
Hypertrophic cardiomyopathy	1	1	2
Sudden unexplained death	3	4	7
Congenital coronary anomalies	5	0	5
Congenital heart disease	2	1	3
Fatty heart	2	1	3
Cardiac conduction system diseases	1	2	3
Viral myocarditis	1	0	1
Rheumatic heart disease	1	0	1
**Total**	**32**	**33**	**65**

The 65 confirmed cases were divided into two age groups by the cut point of 35 years old into ≤35 years old and >35 years old. The differences between these two groups are obvious. The leading diagnosis in the group ≤35 years old was CM, whereas CAD was the leading diagnosis of death in the other group. The distributions of forensic pathological diagnoses in the two groups are illustrated in Table 3.

### The pathology of coronary atherosclerotic heart disease

Among the 65 sport-related SCD cases, 28 cases were identified as CAD. The average age of victims with CAD was (43.35 ± 12.70) years old while the average age of victims without CAD was (30.30 ± 12.79) years old. There were only two cases of CAD in which the victims were under 20 years old (14 and 18 years old). The male-to-female ratio was 8.3:1. CAD accounted for 63.64% in the group of above 35 years old, while only accounted for 21.88% in the group of younger than 35 years old (including 35 years old).

As for the extent of coronary artery stenosis, there was no case in grade I lesions, three cases (10.71%) in grade II lesions, eight cases (28.57%) in grade III lesions and 17 cases (60.71%) in grade IV lesions. Regarding the three main branches of the coronary artery, all 28 cases (100%) exhibited atherosclerosis in the left anterior descending (LAD) artery, six cases (21.43%) exhibited atherosclerosis in the left circumflex artery (LCX) and 10 cases (35.71%) exhibited atherosclerosis in the right coronary artery (RCA). Furthermore, singlebranch atherosclerosis occurred in 14 cases, two branches were involved in 12 cases and all branches were involved in two cases. The cases were divided into the following four types: Type LAD (i.e. disease involving a single artery), Types (LAD + LCX) and (LAD + RCA) (i.e. diseases involving two arteries) and Type (LAD + LCX + RCA) (i.e. disease involving all three arteries). The atherosclerotic pathological distributions of the three branches between the different age groups were analysed, and the results are presented in Table 4.

**Table 4.** The atherosclerotic pathological distribution of the three branches between the different age groups (case number).

Branches	Age ≤35 years old	Age >35 years old
LAD	4	10
LAD + RCA	1	7
LAD + LCX	2	2
LAD + LCX + RCA	0	2

LAD, left anterior descending artery; RCA, right coronary artery; LCX, left circumflex artery.

### The height and cardiac characteristics of 65 sport-related SCD

To study the height and the cardiac characteristics of sport-related SCD, 65 cases of those died from accidental events were included and were matched with the 65 sport-related SCD cases in gender, age and year of death. Based on systematic and thorough autopsy examinations, the average value of height, heart weight, left ventricular wall thickness, right ventricular wall thickness, circumference of tricuspid valve, pulmonary valve, bicuspid valve and aortic valve in the 65 sport-related SCD case and the 65 disease-free accident events death cases are illustrated in Table 5. The average value of height, the heart weight, left ventricular wall thickness, right ventricular wall thickness and circumference of tricuspid valve in the 65 sport-related SCD were larger than that of the 65 disease-free accident events death cases. The results of the Mann-Whitney *U*-test showed statistically significant differences in heart weight and left ventricular wall thickness between the two groups.

**Table 5.** The height and cardiac characteristics between different groups ($\bar{X}$ ± s).

Anatomy index (unit)	Sport-related SCD cases (*n* = 65)	Disease-free accident events death cases *(n* = 65)
Height (cm)	166.63 ± 8.36	166.06 ± 7.32
Heart weight (g)	410.62 ± 133.20^a^	329.68 ± 71.86
Left ventricular wall thickness (cm)	1.33 ± 0.29^b^	1.21 ± 0.21
Right ventricular wall thickness (cm)	0.35 ± 0.11	0.32 ± 0.09
Circumference of tricuspid valve (cm)	11.85 ± 1.62	11.69 ± 1.06
Circumference of pulmonary valve (cm)	7.40 ± 1.16	7.42 ± 0.86
Circumference of bicuspid valve (cm)	9.17 ± 1.34	9.19 ± 1.06
Circumference of aortic valve (cm)	6.64 ± 1.00	6.72 ± 0.78

Versus the group of disease-free accident events death cases: ^a^*P <* 0.05, ^b^*P <* 0.01.

## Discussion

Sport-related SCD has attracted great social attention globally, but studies on this topic are rare in China at the moment. Also is the fact that the current main research concerns are the young athletes, ignoring that this tragic event happens even more in the general population. This is the first study to report the aetiology distribution and basic epidemiology data of sport-related SCD cases (including both young athletes and the general population) in Southern China from the perspective of forensic identification.

The incidence of sudden death (SD) in the competitive athletes ranged from 0.4 to 2.3 per 100 000 people per year [3,5,6,13–15]. However, the incidence of leisure sport-related SD in the general population remains sparse. In France, the overall burden of sport-related SD was 4.6 cases per million people per year, with 6% of cases occurring in young competitive athletes [9]. As for the percentage of sport-related SCD out of all SCD cases, in the present study, the proportion was 3.93%. This result may still be underestimated, and the obvious low number of sport-related SCD in Southern China may be the result of two reasons: one is that some cases lack precise sport-related description and cannot be included in this study, and the other is that our Medicolegal Expertise Center mainly deals with medical dispute cases, and most sport-related SCD cases do not belong to the medical dispute cases and are usually processed directly by the forensic department of police station.

There was a significant gender difference in sport-related SCD cases, and several articles reported that the male-to-female ratio for SD in competitive or daily physical leisure activities ranged from 3:1 to 32:1. [5,7,8,11,16,17]. We previously reported that male-to-female ratio in SCD cases was 4.3:1 [18], and interestingly, in this series the ratio in sport-related SCD cases was even higher (7.1:1). This gender difference can be attributed to several factors: (1) the total number of men participating in the sport is different from that of the women; (2) the sports duration and intensity differ between men and women; (3) the potential disparities may involve gender differences in different vulnerable substrate (underlying structural or electric heart disorder), triggers, and autonomic modulators. For example, the incidence of cardiac disorders predisposing to SCD, such as CAD or HCM, is different between male and female.

The average age of sport-related SD varied in different populations. In competitive athletes, the average age ranged from 18 to 28 years old [5–7,16], whereas in the general population, it ranged from 30 to 46 years old [8,10,11], most frequently in middle-aged men. In our study, the average age of sport-related SCD cases was (35.92 ± 14.23) years old with a peak at 31–45 years old. This difference of SCD in mean age between competitive athletes and the general population may be multivariate. First, the average age of overall competitive athletes seems younger than that of the general population. Second, competitive athletes practise higher intensity exercise than the general population, which forms the basis of SCD. In a recent study, Chappex et al. [11] reported that the average age of sport-related SCD in general population (30.5 years old) was younger than that of non-sport-related SCD in general population (38.2 years old), suggesting that sport plays a role in the occurrence of SCD. However, the mechanism of how sports trigger SCD needs further studies. Third, the fact that sport-related SCD among general population occurs mainly at the middle age may be associated with the progression of CAD.

The types of sports in which the SCD victims participated varied across different countries. Soccer, running and cycling were reported to be the main sports associated with SCD [5–8,10,11,16]. According to our study, heavy physical labour, running and cycling were the leading sports types of SCD. This difference can be attributed to the fact that many people in Guangdong Province have jobs that are physically demanding, and running is the most common and available sport for people to perform. As for running, it has been reported that marathons and half-marathons are associated with a low overall risk for cardiac arrest and SCD [19], and marathon is a risk factor for cardiac arrest [20]. However, in our series, most of the running cases were sprint, supporting that short-distance fast running may also be a risk factor for SCD. The age of SCD victims in different sports varied as well. However, whether the age of SCD victims is related to the type of sport, and whether there is a difference in the mechanisms between different sports in causing SCD remains to be further investigated.

The identification of death causes of sport-related SCD cases is based on the combination of forensic pathological diagnoses with the circumstance before death. Several studies regarding the sport relationship with SD in competitive athletes were reported that SCD was the most frequent forensic diagnosis in competitive athletes. In the USA, HCM was the most frequent diagnosis of SD, followed by coronary development anomalies and myocarditis [6,15]. In the UK [7], idiopathic left ventricular hypertrophy (ILVH) was the most prevalent, followed by SUD and ARVC. In Italy [16], ARVC was the leading diagnosis of sport-related SD, followed by CAD and coronary development anomalies, whereas in France [9] and Spain [10], HCM was the leading one in sport-related SD.

In our series, there were two sport-related SCD cases in total in competitive athletes (one was RCM and the other was ARVC). This low number can be attributed to the fact that the majority of victims of competitive athletes’ SD are sent into the hospital and processed by the department of forensics in a Police Station in China directly as aforementioned. Further studies are still needed for further understanding of SCD in competitive athletes in China. It was reported that CAD was the leading diagnosis of leisure sport-related SD in the general population [10,11,14,21]. Besides, CAD was reported to be proportional to the age and most commonly occurs in victims over 35 years old.

Consistent with these data, in our series, CAD (28 cases) was the principal diagnosis of sport-related SCD followed by CMs (14 cases) and SUD (seven cases). When the 65 SCD cases were divided into two groups (≤35 years old and >35 years old), the forensic diagnoses of sport-related SCD showed a predominance of CAD (21 cases) followed by both SUD (four cases) and CMs (four cases) in the group of >35 years old, and a predominance of CMs (10 cases) followed by CAD (seven cases) and coronary development anomalies (five cases) in the other age group (<35 years old). What deserves special attention is that there were two CAD cases with an age of 14 and 18 years old, respectively; this may be explained by the possibility of a congenital hypercholesterolaemic condition. In addition, the forensic diagnoses of SCD were various within the group of ≤35 years old.

SUD was another major forensic diagnosis of sport-related SCD cases. In our series, SUD accounted for 10.77% of sport-related SCD cases. Suárez-Mier et al. [10] reported that SUD was observed in 11.3% of 168 sport-related SD cases with ages under 35 years old. However, in our study, there were three cases in the group of ≤35 years old and four cases in the group of >35 years old. Two of the four SUD cases in >35 years old group were around 35 years old, while the other two cases were 45 and 60 years old. The characteristic of SUD cases is that there is no identified pathological finding in these victims after systematical autopsy examination. It has been suggested that the primary electrical abnormalities, such as long QT syndrome and Brugada syndrome, are likely to be involved in the pathogenesis of those sport-related SUD cases [22]. However, to identify whether these sport-related SUD cases are associated with these primary electrical abnormalities, molecular studies on blood samples are necessary [23], especially in those cases otherwise described as “cause of death unexplained” or “no pathological findings”. Unfortunately, these samples are rarely available as they are not stored during the autopsy procedure in most cases in China. Our group has focused on the molecular mechanism of SUD in recent years, anticipating collaboration with the department of forensics in the police station, to obtain more cases (including blood samples) for further study and investigation of this issue.

Further analyses of the CAD cases showed that the incidence of sport-related SCD cases increased with increasing coronary artery stenosis severity. Among the three main branches of the coronary artery in CAD cases, LAD was most prone to atherosclerosis (28 cases, 100%) followed by RCA (10 cases, 35.71%) and LCX (seven cases, 25.00%). This finding in sport-related SCD is consistent with our previously reported data [18] and the research conducted in other countries [24]. Regarding the numbers of involved main branches of coronary artery of the CAD cases, a single-branch involvement (14 cases, 50.00%) was the most prevalent followed by two-branch (12 cases, 42.86%) and three-branch (two cases, 7.14%) involvement. Moreover, the branch involved in the entire 14 single-branch CAD cases mentioned above was LAD, suggesting that LAD atherosclerosis may be a risk factor of sport-related SCD, and whether LAD atherosclerosis is a risk factor of sport-related SCD requires further study. When all the 28 CAD cases were divided into two groups (≤35 years old and >35 years old), the differences in the atherosclerotic pathological distributions of the three branches of the two groups showed a non-statistically significant trend towards age (Table 4), and our data support the fact that age is a significant risk factor for coronary atherosclerosis, which has been revealed by other studies [25]. Yet, larger data are needed for deeper analysis.

Compared with the group of 65 disease-free accident-induced deaths, the heart weight and the left ventricular wall thickness of the 65 sport-related SCD cases were larger, and the result showed an obvious statistically significant difference between the two groups. The mean value of heart weight in our group is very close to the average value of heart weight ((406.61 ± 102.55) g, 31 cases) reported by Fornes et al. [21]. However, Holst et al. [5] reported 15 cases in sport-related SCD in competitive athletes with a mean value of (473.57 ± 96.55) g in heart weight in Denmark. This difference between the 65 sport-related SCD cases in our group and the 15 sport-related SCD cases in Holst’s group can be attributed to two factors: First, there are different physical conditions between the general population and competitive athletes. Second, there may be an ethnic difference between Chinese and Danish populations. Similar data are needed for comparison, but unfortunately, today these studies are rarely available. To conclude, it suggests that heart weight is associated with sport-related SCD, and may be a risk factor for sport-related SCD in our series. As for parameters of the heart, only the result of the left ventricular wall thickness showed a statistically significant difference between the two groups. Although there was no significant statistical difference in other parameters, further large data are needed to study whether these parameters of heart are associated with sport-related SCD.

## Conclusion

The incidence of sport-related SCD cases in the general population was more common than we had expected, indicating the need to highlight the risk factors, and provide a prevention strategy to the public. CAD was the leading forensic diagnosis death followed by CM and SUD in all cases. The male gender and the heart weight may be risk factors for sport-related SCD. A genetic molecular autopsy should be used as a necessary tool for further detection, especially in those cases described as “SUD” or “no pathological findings”.

## Perspective

The exact incidence of sport-related SCD is unknown in China. Given the large population in China, the number of sport-related SCD cases is probably high. So it is necessary to collect and to gather more sport-related SCD data to analyse the accurate incidence and the risk factors. In addition, the number of autopsies performed on SCD victims should be increased, andthe case description should be more detailed to determine the accurate incidence and risk factors.
